# Assessment of Health-Related Quality of Life in Patients with Chronic Heart Failure: A Cross-Sectional Study in Vietnam

**DOI:** 10.7759/cureus.51098

**Published:** 2023-12-26

**Authors:** Hung P Truong, Kha M Nguyen, Hien T Tran, Sy V Hoang

**Affiliations:** 1 Department of Internal Medicine, University of Medicine and Pharmacy at Ho Chi Minh City, Ho Chi Minh City, VNM; 2 Department of Cardiology, Hai Duong Hospital, Hai Duong, VNM

**Keywords:** mental component score, physical component score, heart failure, sf-36, health-related quality of life

## Abstract

Introduction

Heart failure is currently a global health issue, imposing a burden on disease prevalence and mortality rates for patients, while simultaneously impacting the quality of life for affected individuals. Data on assessing the health-related quality of life (HRQoL) of patients with chronic heart failure in developing countries, including Vietnam, is still limited. This study was conducted with the aim of describing the quality of life of patients with chronic heart failure in Vietnam.

Methods

This cross-sectional investigation enrolled 140 chronic heart failure outpatients, utilizing a convenience sample at Hai Duong Province Hospital, Vietnam, spanning from December 2021 to April 2022. Essential patient variables encompassing age, gender, and heart failure duration were gathered. Surveying of patients took place at the outpatient clinic during chronic heart failure follow-up visits using the 36-Item Short Form Health Survey (SF-36) questionnaire. The SF-36 comprises eight dimensions: (1) Physical functioning, (2) Role limitations due to physical health, (3) Bodily pain, (4) General health perceptions, (5) Vitality, (6) Social role functioning, (7) Role limitations due to emotional health, and (8) Mental health. Component analysis of the SF-36 revealed two distinct concepts: a physical component summary (PCS) reflecting the physical aspect and a mental component summary (MCS) reflecting the mental aspect.

Results

The research involved 140 participants diagnosed with chronic heart failure, having a median age of 59 years (interquartile range (IQR): 52-63). Among them, 61.4% were male, and 50% exhibited reduced left ventricular ejection fraction (LVEF) (≤ 40%). The role limitations due to the physical health domain indicated the lowest score, registering a median value of 0 (IQR 0-25). Domains with median scores below the 25-point threshold encompassed role limitations due to physical health (0 points). Those with scores ranging from 25 to 49 points constituted general health perceptions (25 points), role limitations due to emotional health (33.3 points), vitality (45 points), and mental health (48 points). Bodily pain and social role functioning achieved median scores at a moderate level (50-74 points), scoring 62 and 62.5 points, respectively. The overall HRQoL score on the SF-36 scale was 45.2 (IQR: 32.1-58.7) points. Median scores for the PCS and MCS were 44.3 (IQR: 30.5-52) and 47.0 (IQR: 32.6-65.4), respectively. No statistically significant differences in PCS and MCS scores were observed when subgroup analysis was performed based on variables like age, gender, or LVEF. However, in the vitality domain, female patients exhibited a significantly lower median score than male patients (p-value = 0.046). In the physical functioning domain, individuals aged ≥ 60 had lower median scores than those aged < 60 years (p = 0.022). Additionally, the group with LVEF ≤ 40% had lower median scores compared to the group with LVEF > 40% (p = 0.038) in role limitations due to emotional health domain.

Conclusion

In Vietnam, the HRQoL in the outpatient population with chronic heart failure was notably low when assessed using the SF-36 questionnaire. Large-scale, multicenter studies are needed to provide stronger, more conclusive evidence.

## Introduction

Heart failure, a pressing public health issue, significantly compromises the well-being of individuals, extending its impact beyond the physical realm to emotional and social dimensions. The challenges of symptom management and treatment adherence further underscore its pervasive influence on patients' lives. Addressing the multifaceted consequences of heart failure is crucial for devising effective interventions that not only enhance clinical outcomes but also improve the overall quality of life for those affected [1]. In Vietnam, the rising prevalence of heart failure emerges as a pressing concern, correlating closely with the demographic shift towards an aging population. This increase in heart failure incidence not only underscores the growing healthcare burden but also emphasizes the imperative for tailored strategies and interventions to address the unique challenges posed by an aging demographic. Recent data indicates that the prevalence of heart failure in Southeast Asia is comparable to the global average. Information from the 2019 European Society of Cardiology (ESC) Heart Failure Association (HFA) atlas shows a prevalence of 1.7% for heart failure [2]. The prevalence rates for heart failure in the United States, United Kingdom, Indonesia, the Philippines, and Thailand are 2.5%, 1.6%, 5%, 1-2%, and 0.4%, respectively [2]. Heart failure is responsible for nearly 20% of hospitalizations in the Southeast Asian region, with readmission rates within 30 days reaching up to 15%. Specifically, the readmission rates are around 8% in Indonesia, Malaysia, and Vietnam, and approximately 15% in Taiwan [2]. Importantly, heart failure significantly impacts a patient's health-related quality of life (HRQoL) causing a decline in both the physical and mental well-being of the individual [3].

Studies in Western countries show that patients with heart failure experience a significantly higher frequency of deterioration in HRQoL compared to the frequency of mortality caused by this condition [4,5]. Additionally, treatment guidelines for heart failure emphasize the importance of patients' quality of life as a standard criterion for care. Despite outstanding advances in treatment, the HRQoL among patients with chronic heart failure remains relatively poor worldwide, resulting in a high social and economic burden, particularly in low- and middle-income countries. The reality of HRQoL of heart failure patients will provide a more multidimensional perspective on the effectiveness of treatment strategies, as well as local medical policies [6,7].

Several tools and scales are available for assessing the quality of life of patients with heart failure, such as the Kansas City Cardiomyopathy Questionnaire (KCCQ), 36-Item Short Form Health Survey questionnaire (SF-36), and EQ-5D-5L. The SF-36 is a widely utilized instrument for assessing HRQoL and is employed globally, having been translated into various languages. Subsequently, the questionnaire has been translated into 120 languages and has been used globally to assess HRQoL in various countries (e.g., Vietnam, China, India, The Netherlands, and Spain) [8,9].

Despite the increasing prevalence of chronic heart failure in Vietnam, there have been few published reports about the HRQoL in this population. In 2018, Tran et al. published a report on the HRQoL among patients with cardiovascular diseases in Vietnam [10]. The cardiovascular diseases primarily discussed were hypertension and coronary artery disease. In 2021, another author from Vietnam assessed the HRQoL in patients with congenital heart disease [11]. Clearly, as of the current time, there is no published study specifically evaluating the HRQoL for heart failure patients. Therefore, this study was conducted with the aim of assessing the HRQoL in Vietnamese patients undergoing outpatient treatment for chronic heart failure using the SF-36 questionnaire. This will provide data on the current status of HRQoL in outpatient chronic heart failure patients in Vietnam.

## Materials and methods

Study design and population

This cross-sectional descriptive study was conducted at the Cardiology Center, Hai Duong Provincial Hospital, Vietnam, from December 2021 to April 2022. The study population consisted of patients diagnosed with chronic heart failure who received outpatient treatment. The inclusion criteria were as follows: (i) age 18-65 years, (ii) diagnosis with chronic heart failure according to 2021 ESC Guidelines for the diagnosis and treatment of acute and chronic heart failure [12] and currently undergoing outpatient treatment, (iii) alert, demonstrating a clear understanding of the content of the questions in the SF-36 questionnaire, and (iv) willing to consent to participate in the study. The exclusion criteria included patients with cognitive disorders, those unable to respond, those experiencing difficulty in recall or communication such as hearing or language impairment, or failure to complete the questionnaire in its entirety. The study was approved by the Ethics Committee for Biomedical Research at the University of Medicine and Pharmacy, Ho Chi Minh City (approval number: ID:835/HDDD-DHYD dated December 20, 2021).

In this study, a convenient sampling method was employed. Patients were surveyed using the SF-36 questionnaire at the outpatient clinic, solely at the time of follow-up visits for chronic heart failure during the study period. All outpatients with chronic heart failure receiving treatment at Hai Duong Provincial Hospital during the study period and meeting the inclusion criteria were included in the research.

Study variables

The anthropometric and clinical variables of the patients were collected, including age, gender, duration of heart failure, and left ventricular ejection fraction. The questionnaire used to assess HRQoL was a Vietnamese version of the SF-36. This questionnaire has been authorized by the author of the Vietnamese version to be used for research purposes [8,13]. The instrument comprises 36 items with two composite measures of physical and mental components that include the following eight domains [14]: (1) physical functioning (PF), (2) role limitations due to physical health (RP), (3) bodily pain (BP), (4) general health perceptions (GH), (5) vitality (VT), (6) social role functioning (SF), (7) role limitations due to emotional health (RE), and (8) mental health (MH). Each item was scored from 0 to 100, with higher scores indicating better quality of life. Participants were classified into four groups depending on their HRQoL scores. Scores from 0 to 25 were considered very low, scores from 25 to < 50 were low, scores from 50 to < 75 were moderate, and scores between 75 to 100 were high [15,16]. The physical component score is calculated by summing scores from four domains: PF (10 items), RP (four items), BP (two items), and GH (five items). The mental component score is calculated by summing scores from four domains: RE (three items), VT (four items), SF (two items), and MH (five items)[14].

Statistical analysis

Data entry and processing were conducted using Stata Statistical Software 14.2 (2015; StataCorp LP, College Station, Texas, United States) on a Windows operating system (Microsoft Corporation, Redmond, Washington, United States). The normality check of the numerical variables was performed using the Shapiro-Wilk test. Continuous variables with a normal distribution were described as mean ± standard deviation. If the distributions were not normal, they were described using the median (25th-75th quartiles). Categorical and ordinal variables are defined as frequencies and percentages. Differences in means between groups were compared using the t-test for normally distributed variables and the Mann-Whitney U test for non-normally distributed variables. Differences in the frequency distributions of categorical variables were assessed using the chi-square test (χ²). Statistical significance was defined as a p-value < 0.05, indicating statistical significance.

## Results

Baseline characteristics

We enrolled 140 patients with chronic heart failure who met the inclusion criteria. The median age of the participants was 59 years (range 52-63), with a higher proportion of male patients (61.4%, n=86). The group of patients under 60 years of age accounted for 54.3%. The median duration of heart failure in the study was five years, with an interquartile range (IQR) of 2-16. 

Table 1 gives a comprehensive snapshot of the HRQoL among outpatients with chronic heart failure. The domains with the lowest scores included RP, which scored only 0 points. Domains with median scores falling into the low-level range (25-49 points) included GH (25 points), RE (33.3 points), VT (45 points), and MH (48 points). BP and SF obtained median scores in the moderate level range (50-74 points), with scores of 62 and 62.5 points, respectively. This study showed that the MCS was higher than the PCS. The overall HRQoL score on the SF-36 scale was 45.2 (IQR 32.1-58.7) points. 

**Table 1:** Scores for each domain component and the total score of the 36-SF questionnaire (N = 140) IQR: interquartile range; 36-SF: 36-Item Short Form Health Survey; HRQoL: health-related quality of life

Domain	Median (points)	IQR (25^th^ – 75^th^)
Physical functioning (PF)	65	55-75
Role limitations due to physical health (RP)	0	0 -25
Bodily pain (BP)	62	42-74
General health perceptions (GH)	25	15-40
Vitality (VT)	45	35 – 55
Social role functioning (SF)	62.5	37.5-75
Role limitations due to emotional health (RE)	33.3	0-91.7
Mental health (MH)	48	44-60
Physical Component Score (PCS)	44.3	30.5–52
Mental Component Score (MCS)	47.0	32.6–65.4
Overall HRQoL score	45.2	32.1 – 58.7

**Figure 1:**
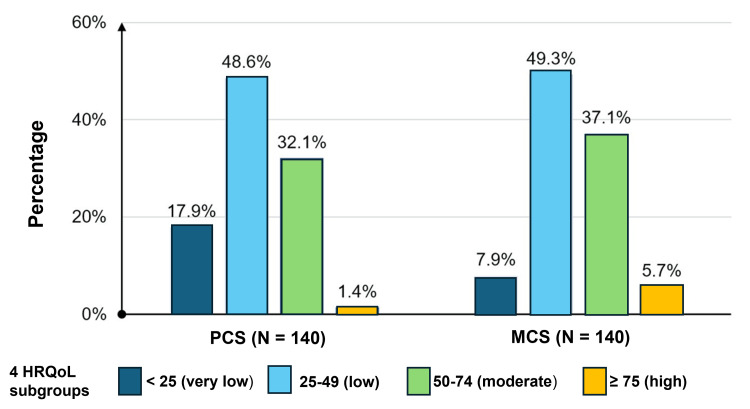
The distribution in the PCS and MCS across four HRQoL subgroups PCS: physical component summary; MCS: mental component summary; HRQoL: health-related quality of life

When describing the two components, PCS and MCS, across the four HRQoL subgroups, the proportion of patients with low quality of life (25-49 points) was the highest, accounting for 48.6% for PCS and 49.3% for MCS. The group of patients with high quality of life in both PCS and MCS components had a low percentage, with rates of 1.4% and 5.7%, respectively (Figure 1).

Table 2 shows the characteristics of scores for PCS and MCS according to gender and HRQoL subgroups. The results revealed that males scored higher than females in all subgroups for both PCS and MCS.

**Table 2:** Distribution of PCS and MCS by gender and HRQoL subgroups PCS: physical component summary; MCS: mental component summary; HRQoL: health-related quality of life The values are expressed as quantities (percentages)

Gender	Total (N = 140)	PCS by 4 HRQoL Subgroups				MCS by 4 HRQoL Subgroups			
		< 25	25 - 49	50 - 74	≥ 75	< 25	25 - 49	50 - 74	≥ 75
Male, n (%)	86 (61.4)	13 (9.3)	45 (32.2)	27 (19.3)	1 (0.7)	6 (4.3)	43 (30.7)	31 (22.1)	6 (4.3)
Female, n (%)	54 (38.6)	12 (8.6)	23 (16.4)	18 (12.8)	1 (0.7)	5 (3.6)	26 (18.6)	21 (15.0)	2 (1.4)

Table 3 gives the comparison of PCS, MCS, and overall HRQoL scores based on the fundamental clinical characteristics of patients in the study. Younger heart failure patients (< 60 years) exhibited higher scores in all three categories compared to older individuals (≥ 60 years). Similarly, patients with more severe heart failure, as indicated by an LVEF < 40%, scored lower in PCS, MCS, and overall HRQoL compared to the group with higher LVEF. However, no significant differences were found among age groups (< 60 years or ≥ 60 years), gender (male or female), and LVEF (ejection fraction (EF) ≤ 40% or EF > 40%) in terms of PCS, MCS, and overall HRQoL scores.

**Table 3:** The distribution of PCS, MCS, and overall HRQoL scores by age, gender, and LVEF * Mann-Whitney U test PCS: physical component summary; MCS: mental component summary; HRQoL: health-related quality of life; LVEF: left ventricular ejection fraction

Characteristics		Median (IQR)	p-value
Physical component score			
Age	< 60 years	46.4 (31.8-55.5)	0.104^*^
	≥ 60 years	41.1 (28.6-49)	
Gender	Male	44.8 (31.8-51.8)	0.508^*^
	Female	41.4 (28-52.3)	
LVEF	EF ≤ 40%	43.9 (31.8-50.5)	0.439^*^
	EF > 40%	44.5 (29.3-53.8)	
Mental component score			
Age	< 60 years	49.1 (31.9-66.7)	0.308^*^
	≥ 60 years	44.5 (32.8-57.8)	
Gender	Male	45.6 (33.8-65.4)	0.901^*^
	Female	48.1 (30.6-64.5)	
LVEF	EF ≤ 40%	41.6 (31-61.2)	0.064^*^
	EF > 40%	50 (34.5-66.4)	
Overall HRQoL score			
Age	< 60 years	48.7 (34.2-59.9)	0.172^*^
	≥ 60 years	42.9 (31.2-51.2)	
Gender	Male	45.6 (33.1-59.4)	0.593^*^
	Female	43.5 (28.9-57.5)	
LVEF	EF ≤ 40%	44.5 (32.1-57.0)	0.205^*^
	EF > 40%	47.2 (34-61.1)	

Table 4 illustrates the HRQoL scores for the eight main domains in the 36-SF questionnaire. The findings showed no significant differences in HRQoL scores within each domain between age groups (<60 years or ≥60 years), gender, and LVEF (EF ≤ 40% or EF > 40%). However, the PF domain was significantly lower in the age ≥60 group (p = 0.022), the VT domain was lower in females (p = 0.046), and RE was lower in the LVEF ≤ 40% group (p = 0.038).

**Table 4:** Distribution of scores for the eight domains by age, gender, and LVEF Values were presented as Mean ± Standard Deviation or Median (Interquartile range) *Normal distribution; ^a ^chi-square test (χ²); ^b ^Man Whitney U test LVEF: left ventricular ejection fraction

Domain	Age (years)		p-value	Gender		p-value	LVEF (%)		p-value
	< 60	≥ 60		Male	Female		EF ≤ 40	EF > 40	
GH *	28.1 ± 15.9	26.2 ± 16.1	0.463^a^	29 ± 15.6	24.3 ± 16.1	0.062^a^	27.5 ± 17.1	26.9 ± 14.8	0.862^a^
PF	70 (56.3-75)	62.5 (55-75)	0.022^b^	70 (58.8-75)	62.5 (55-75)	0.160^b^	65 (55-75)	65 (55-75)	0.985^b^
RP	0 (0-50)	0 (0-25)	0.063^b^	0 (0-25)	0 (0-50)	0.613^b^	0 (0-25)	0 (0-50)	0.166^b^
RE	33.3 (0-100)	33.3 (0-66.7)	0.247^b^	33.3 (0-66.7)	33.3 (0-100)	0.794^b^	33.3 (0-66.7)	33.3 (0-100)	0.038^b^
SF*	59.9 ± 24.8	53.1 ± 21.9	0.082^a^	57.4 ± 24.5	55.8 ± 22.5	0.623^a^	54.1 ± 25.6	59.5 ± 21.4	0.293^a^
BP	68 (42-74)	62 (42-74)	0.670^b^	62 (42-74)	62 (42-74)	0.625^b^	62 (42-74)	62 (41-74)	0.790^b^
VT	45 (35-55)	45 (35-50)	0.496^b^	45 (35-55)	40 (35-50)	0.046^b^	45 (35-55)	45 (35-55)	0.595^b^
MH	48 (40-60)	52 (44-60)	0.275^b^	50 (44-60)	48 (40-61)	0.969^b^	48 (40-61)	50 (44-60)	0.440^b^

## Discussion

The overall median HRQoL score according to the SF-36 questionnaire in our study population was 45.2 (IQR 32.1-58.7), with the PCS at 44.3, which was lower than the MCS at 47.0. In their study in Saudi Arabia, AbuRuz et al. also found that the HRQoL scores for chronic heart failure patients were generally low, with PCS and MCS scores averaging 36.7 ± 12.4 and 48.8 ± 6.5, respectively, where the MCS was higher than the PCS [15]. In our study, the areas with the least favorable scores encompassed RP, registering a mere 0 points. Domains with median scores falling within the lower range (25-49 points) included GH with 25 points, RE with 33.3 points, VT with 45 points, and MH with 48 points. BP and SF domains achieved median scores within the moderate range (50-74 points), securing scores of 62 and 62.5 points, respectively. Upon conducting subgroup analysis based on variables such as age, gender, or LVEF, no statistically significant differences were observed in PCS, MCS, and overall HRQoL scores.

Alharbi et al. in Saudi Arabia [16] and Rodríguez-Artalejo et al. in Spain [17] also noted that the RP domain had the lowest scores, consistent with our study. Heart failure is characterized by a cluster of symptoms and signs resulting from structural or functional cardiac abnormalities [18]. Heart failure patients experience physical symptoms, including fatigue, dyspnea, chest pain, edema, and sleep difficulties, which adversely impact their ability to perform daily activities. A systematic review and meta-analysis in 2020 by Moradi et al. also found that the RP was the domain with the lowest scores in the SF-36 questionnaire, with a score of 40.5 (14.8-66.2) [19]. However, the scores were still higher than those in our study, and the differences between these studies could be explained by variations in research methods, comorbidities, the duration of heart failure, treatment adherence, and variations in healthcare quality.

Furthermore, our study, along with Alharbi et al. [16] and Rodríguez-Artalejo et al. [17], showed that the SF and BP domains had high scores in the SF-36 assessment. Heo et al. surveyed to understand how heart failure patients define their quality of life. The authors found that heart failure patients strive to maintain a positive psychosocial attitude despite limitations in daily activities, positively affecting HRQoL [4]. In Vietnamese heart failure patients, the support and care of family and the loving social culture may also contribute as essential factors to their well-being.

The median scores of the PCS and MCS were not significantly different among age groups (< 60 or ≥ 60 years), gender, and LVEF (EF ≤ 40% or EF > 40%) (all p-values > 0.05). However, the group with age ≥ 60 years showed significantly lower physical health (PF) compared to the group with age < 60 (p = 0.022). Milanovic et al. have shown that aging is significantly associated with poor progress in the ability to perform physical activities among older adults due to the aging process. This difference may be explained by the fact that older patients experience a reduction in muscle strength and muscle mass distribution in both upper and lower extremities and changes in body fat percentage, flexibility, agility, and endurance [20]. This suggests that older heart failure patients (≥ 60 years) may have lower scores in the PF domain.

Female patients had significantly lower scores in the VT domain compared to male patients (p = 0.046). Several studies have found that males tend to have better mental adjustment abilities. Möller-Leimkühler et al. investigated the association of gender with depression and cardiac diseases [21]. The authors observed that women were more strongly affected by the psychological stress related to cardiac diseases and depression and the direct and indirect impacts of severe psychological stress compared to men. This difference in mental well-being between men and women is attributed to the more decisive influence of psychological stress factors in women due to alters in the functioning of the hypothalamic-pituitary-adrenal axis and the autonomic nervous system. Alharbi et al. in Saudi Arabia also noted that men had higher scores in the MCS compared to women (p < 0.001) [16]. In our study, patients with heart failure and LVEF ≤ 40% had significantly lower scores in the RE domain compared to those with LVEF > 40% (p = 0.038). This suggests that heart failure patients with reduced LVEF experience more challenges in daily activities due to their psychological health.

A study by Bekfani et al. also investigated psychological and mental factors in patients with heart failure with reduced EF (HFrEF) and preserved EF (HFpEF) [22]. The study found that the HFpEF group had lower MCS, VT scores, elevated anxiety, and increased depression scores compared to the HFrEF group. However, there was no difference in the RE domain between HFpEF, HFrEF, and non-HF controls (p = 0.08). It is important to note that Bekfani's study had a limited population of n=55. The authors suggested larger sample size studies are needed to confirm these results. The differences between the studies could be attributed to variations in the diagnosis of HFpEF and the influence of the coronavirus disease 2019 (COVID-19) pandemic, which might affect the assessment of psychological health domains in our study.

Regular assessments of HRQoL may help identify patients with a low quality of life and assist clinical physicians in focusing on the primary concerns the patient needs to address. Furthermore, it serves as a tool to assess the impact of various treatment interventions on the progression of the disease. It is also necessary to identify the factors that influence the quality of life in heart failure patients to manage and improve their quality of life effectively [6].

Our study has several limitations. Firstly, we conducted a single-center study during the COVID-19 pandemic with a relatively small sample size and a short study duration, which may have introduced confounding factors. Secondly, we did not evaluate the association of SF-36 scores with outcomes such as mortality or hospitalization in heart failure patients. Thirdly, we did not investigate various factors, such as comorbidities, sleep quality, and the time of heart failure diagnosis, that may be related to the quality of life in heart failure patients. Additionally, issues related to optimizing heart failure treatment through recommended medications or devices also significantly impact the quality of life of chronic heart failure patients. This study solely describes the quality of life characteristics of chronic heart failure patients using the SF-36 questionnaire in a province in Vietnam. In the future, it is essential to conduct larger, multicenter studies with long-term follow-ups, as well as comprehensive assessments of all relevant factors, to provide a more holistic perspective on the quality of life of chronic heart failure patients in Vietnam.

## Conclusions

In Vietnam, the HRQoL among outpatients with chronic heart failure is markedly diminished when evaluated through the SF-36 questionnaire. The PCS, MCS, and overall HRQoL scores showed no significant differences when compared across subgroups based on fundamental clinical parameters. Robust, multicenter studies are imperative to provide more compelling and conclusive evidence, as well as to comprehensively assess the various factors that may influence the evaluation of HRQoL in patients with chronic outpatient heart failure.

## References

[1] Savarese G, Lund LH (2017). Global public health burden of heart failure. Card Fail Rev.

[2] Shahim B, Kapelios CJ, Savarese G, Lund LH (2023). Global public health burden of heart failure: an updated review. Card Fail Rev.

[3] Salyer J, Flattery M, Lyon DE (2019). Heart failure symptom clusters and quality of life. Heart Lung.

[4] Heo S, Lennie TA, Okoli C, Moser DK (2009). Quality of life in patients with heart failure: ask the patients. Heart Lung.

[5] Fotos NV, Giakoumidakis K, Kollia Z, Galanis P, Copanitsanou P, Pananoudaki E, Brokalaki H (2013). Health-related quality of life of patients with severe heart failure. A cross-sectional multicentre study. Scand J Caring Sci.

[6] Do TN, Do QH, Cowie MR (2019). Effect of the optimize heart failure care program on clinical and patient outcomes - the pilot implementation in Vietnam. Int J Cardiol Heart Vasc.

[7] Kim SE, Yoo BS (2023). Treatment strategies of improving quality of care in patients with heart failure. Korean Circ J.

[8] Watkins RE, Plant AJ, Sang D, O'Rourke T, Gushulak B (2000). Development of a Vietnamese version of the short form-36 health survey. Asia Pac J Public Health.

[9] Ghaddar A, Mateo I, Sanchez P (2008). Occupational stress and mental health among correctional officers: a cross-sectional study. J Occup Health.

[10] Tran BX, Moir MP, Thai TP (2018). Socioeconomic inequalities in health-related quality of life among patients with cardiovascular diseases in Vietnam. Biomed Res Int.

[11] Truong TH, Kim NT, Nguyen MT, Do DL, Nguyen HT, Le TT, Le HA (2021). Quality of life and health status of hospitalized adults with congenital heart disease in Vietnam: a cross-sectional study. BMC Cardiovasc Disord.

[12] McDonagh TA, Metra M, Adamo M (2021). 2021 ESC Guidelines for the diagnosis and treatment of acute and chronic heart failure. Eur Heart J.

[13] Vo KT, Nguyen KT (2017). Translation, cultural adaptation and preliminary validity of the Vietnamese short form 36 (SF 36). Vietnam J Diabetes Endocrinol.

[14] Ware JE Jr, Sherbourne CD (1992). The MOS 36-item short-form health survey (SF-36). I. Conceptual framework and item selection. Med Care.

[15] AbuRuz ME, Alaloul F, Saifan A, Masa'deh R, Abusalem S (2015). Quality of life for Saudi patients with heart failure: a cross-sectional correlational study. Glob J Health Sci.

[16] Alharbi M, Alharbi F, AlTuwayjiri A (2022). Assessment of health-related quality of life in patients with heart failure: a cross-sectional study in Saudi Arabia. Health Qual Life Outcomes.

[17] Rodríguez-Artalejo F, Guallar-Castillón P, Pascual CR (2005). Health-related quality of life as a predictor of hospital readmission and death among patients with heart failure. Arch Intern Med.

[18] Bozkurt B, Coats AJ, Tsutsui H (2021). Universal definition and classification of heart failure: a report of the Heart Failure Society of America, Heart Failure Association of the European Society of Cardiology, Japanese Heart Failure Society and Writing Committee of the Universal Definition of Heart Failure: Endorsed by the Canadian Heart Failure Society, Heart Failure Association of India, Cardiac Society of Australia and New Zealand, and Chinese Heart Failure Association. Eur J Heart Fail.

[19] Moradi M, Daneshi F, Behzadmehr R, Rafiemanesh H, Bouya S, Raeisi M (2020). Quality of life of chronic heart failure patients: a systematic review and meta-analysis. Heart Fail Rev.

[20] Milanović Z, Pantelić S, Trajković N, Sporiš G, Kostić R, James N (2013). Age-related decrease in physical activity and functional fitness among elderly men and women. Clin Interv Aging.

[21] Möller-Leimkühler AM (2010). Higher comorbidity of depression and cardiovascular disease in women: a biopsychosocial perspective. World J Biol Psychiatry.

[22] Bekfani T, Nisser J, Derlien S (2021). Psychosocial factors, mental health, and coordination capacity in patients with heart failure with preserved ejection fraction compared with heart failure with reduced ejection fraction. ESC Heart Fail.

